# Obituary

**Published:** 1848-08

**Authors:** 


*Obituary.*—Died in this city, on the 26th ult. Dr. Charles Schmidt, aged 24 years. Dr. Schmidt was a German, educated at the school of Giessen. He established himself here as a practitioner of medicine a year since, and was much esteemed by his medical and other acquaintances. The disease of which he died was Typhoid fever.

